# Role of non-governmental organizations in moving toward universal health coverage: A case study in Iran

**DOI:** 10.3389/fpubh.2022.985079

**Published:** 2022-10-20

**Authors:** Arman Sanadgol, Leila Doshmangir, Rahim Khodayari-Zarnaq, Vladimir Sergeevich Gordeev

**Affiliations:** ^1^Department of Health Policy and Management, Tabriz Health Services Management Research Center, School of Management and Medical Informatics, Tabriz University of Medical Sciences, Tabriz, Iran; ^2^Student Research Committee, Tabriz University of Medical Sciences, Tabriz, Iran; ^3^Social Determinants of Health Research Center, Tabriz University of Medical Sciences, Tabriz, Iran; ^4^Wolfson Institute of Population Health, Queen Mary University of London, London, United Kingdom; ^5^Department of Infectious Disease Epidemiology, London School of Hygiene and Tropical Medicine, London, United Kingdom

**Keywords:** non-governmental organizations, universal health coverage, services coverage, population coverage, financial coverage, health system research and policy

## Abstract

**Background:**

Delivering essential health services through non-governmental organizations (NGOs) could facilitate moving toward universal health coverage (UHC), especially in low- and middle-income countries. This study investigates the viewpoints of Iranian health system experts and executive stakeholders on the role of NGOs in moving toward UHC.

**Method:**

We conducted 33 semi-structured interviews with health policymakers, NGO representatives at the national and provincial level, and other key informants and analyzed using content analyses methods, using MAXQDA 12. The inductive-deductive approach was used for qualitative data analyses.

**Result:**

Based on the thematic analysis of interviews and document reviews, nine main themes and one hundred and five sub-themes were identified. Each theme was categorized based on NGO-, society-, and government-related factors.

**Conclusion:**

Recognizing the critical role of NGOs and their contribution in moving toward UHC is essential, particularly in the local context. Collaboration between NGO stakeholders and the government could facilitate moving toward UHC.

## Introduction

Universal health coverage (UHC) is essential to achieve optimal health and justice in every country (1), and all community members should have access to all aspects of health services, including prevention, treatment, and rehabilitation (2). Health systems need to be sufficiently efficient in population, service, and financial coverage to achieve sustainable UHC (3). However, despite injecting financial and human resources into the health systems, many countries still have difficulties achieving UHC (4, 5), as allocating funding for healthcare priorities alone is insufficient (6).

Like many other countries, Iran has made additional efforts to move toward UHC during the last decades. Recent reforms aimed to improve the situation by increasing the number and variety of services provided to patients (especially to the poor) and increasing access to primary healthcare in remote parts of the country (4). However, providing adequate and fair services to all populations remains one of the main challenges in Iran's UHC goals (7). As UHC has become a priority for the global health community, understanding the role of various groups and organizations in the country and how they contribute to reaching this goal is critical. Non-governmental organizations (NGOs) can help achieve the goals of UHC (5). NGOs have grown significantly since the mid-1970s, and through creative activities and increased public participation, they have increased population coverage, especially in disadvantaged and rural communities (8–10). The term “NGO” usually refers to any non-profit voluntary group of global citizens who work locally, nationally, and internationally for various cultural, social, charitable, and professional purposes (11).

### NGO activities in Iran

In Iran, NGO is an organization that is legal, non-profitable, independent from the government or any political or religious organization, and voluntary and is known as “SAMAN,” a short version of the Persian phrase “Sazmanha-ye Mardom Nahad,” translated as “community-based organizations” or “people's organizations.” NGOs have similar functions to Charites. According to the NGO functions, government institutions such as Ministry of Interior, Governor and Governorates, Ministry of Sports and Youth, Law Enforcement Command, and Welfare Organization monitor the activities of NGOs in Iran (12). In 2017, National Health Assembly was funded consisting representatives from different policy and management levels and health-related NGOs (13). In addition, three national assembly participate in the process of health-related policies including the National Assembly of Youth NGO, the National Assembly of People's organizations, and the Assembly of Marriage Donors. Also, there are foreign NGOs that do not have an impact on Iranian health policy, and their main focus is on the immigrants who enter Iran. The Iranian Diabetes NGO, which provides disease-related services, or the Iranian AIDS NGO, which is responsible for organizing and coordinating all public activities in the field of HIV/AIDS, are the popular NGOs in Iran. Following the last official report based on 2004 Census, there were 6,914 active NGOs in Iran. According to unofficial statistics (Deputy Minister of Social Participation), the number of NGOs was about twenty thousand in 2020. Since the beginning of NGOs' establishment in Iran, they have had a role in healthcare provision, treatment and pharmaceutical activities, financial support, education and prevention, social health, consulting role, and technical information, as well as in monitoring individuals' communities' access to healthcare (14, 15). Furthermore, new adopted policies and regulations support public organizations and NGOs (i.e., articles 95, 97, 104, 140, and 163 of the Law of the Iran Fourth Economic, Social, and Cultural Five-Year Development Plan the Horizon of Iran 1404). NGO contributions to universities of medical sciences in Iran were around 357 to 405 million USD in 2016–2017 (16). Until 2015, about 6 billion USD channeled through NGOs' donations supported the construction of hospitals, equipment purchasing, and expanded healthcare service delivery infrastructure. Overall, some NGOs have a pivotal role in providing financial assistance for the construction and equipment of the hospitals.

### UHC in Iran

The framework Iranian health system has a clear cut three-level design, containing primary, secondary, and tertiary facilities, whose financial resources include government funds, general taxation, health insurance, out-of-pocket, and individual donations. After the revolution, Iran has made many changes in its health system to achieve health for all, and since UHC was considered as the main priority, its components were included in the national upstream policy documents such as Five-Year Development Plans and 20-year vision plan (17, 18). To translate national policy documents to implementation plans, major reforms toward achieving UHC including the Family Physician Program (2004), merging all existing health insurance funds into MSIO (2012) and the Health Transformation Plan(HTP) (2014), were developed in Iran (19, 20). One of the aims of the HTP that the then government emphasized on it was socialization of health. In this regard, civil societies, especially NGOs, were considered as key factor in the health sector functions. In 2017, the National Health Assembly was formed and NGOs officially entered the policymaking processes in Iran's health system (21). Implementing HTP along with other policy interventions led to an impact on the three dimensions of UHC, including providing primary healthcare, promoting health indexes, controlling epidemics, preventing and eradicating some diseases, extending healthcare insurance, improving health resources both qualitatively and quantitatively, improving the quality of healthcare and services, and developing pharmaceutical industry (7). Also, out-of-pocket expenditures have decreased from 80.5 (1995) to 38.8% (2016) of current health expenditure (22).

To develop working policies to engage NGOs in a movement toward UHC effectively, it is essential to understand the potential and roles of NGOs activities in facilitating progress toward UHC goals. This study aimed to explore the views of NGOs' participants, policymakers, and planners on the activities performed by NGOs, examine to what extent they could play a role in progress toward UHC in Iran, and how their function can be maximized in services provision in the long run.

## Materials and methods

### Design and setting

This qualitative case study was carried out using semi-structured interviews and documentary reviews. The study's design and the presentation of findings follow the Consolidated Criteria for Reporting Qualitative Research (COREQ) 32-item checklist (23).

### Participants

Semi-structured interviews were conducted with NGO managers, board of directors and technical experts, faculty members, researchers, scholars, heads of NGOs, and experts of government agencies that issue licenses to NGOs' establishments. Considering the scope and role of NGOs and the geographical extent of Iran, purposeful sampling with maximum variation was used to select participants. In addition to practice backgrounds and experience, for NGOs, criteria such as the level of activity (provincial, national, and international), the number of years of activity, and their willingness to participate were considered. For the rest of the participants, individuals' educational and professional background was also considered when selecting interviewees. People with experience in collaboration with health organizations and involvement in related activities were preferred. Before participation, the prospective participants were contacted and invited to participate in the study *via* email or telephone, and the interview was conducted face to face. Data saturation was considered as the basis for the number of study participants. Interviewee characteristics are presented in Table 1.

**Table 1:** Baseline characteristics of study participants.

**Participant's characteristics**	**Number (%)**
**Age (year)**
≤30	5 (15)
31–40	17 (52)
41–50	7 (21)
>50	4 (12)
**Gender**
Male	14 (42)
Female	19 (58)
**Education**
≤Diploma	3 (9)
BSc^a^	8 (24)
≥MSc^b^	22 (67)
**Job position**
Manager/board of director of NGO' staff	19 (58)
Head of unit	2 (6)
Expert of unit	4 (12)
Researcher and faculty member	8 (24)
**Work experience (year)**
5–10	12 (36)
11–15	11 (33)
>15	10 (31)
**NGOs activities (‘among NGO staff)**
Cancer	2 (10.7)
Phenylketonuria	1 (5.2)
Autism	1 (5.2)
Epilepsy	1 (5.2)
Hemophilia	1 (5.2)
HIV/AIDS	2 (10.7)
Diabetes	1 (5.2)
Thalassemia	1 (5.2)
Multiple sclerosis (MS)	1 (5.2)
Specific diseases	2 (10.7)
Kidney	1 (5.2)
Mental diseases	2 (10.7)
Social health	1 (5.2)
Development of public health	1 (5.2)
Reduce social harms and diseases	1 (5.2)

^a^ Bachelor of science.

^b^ Master of science.

### Interview tool and data collection

A semi-structured interview tool was developed to guide all interviews and comprised five parts (i.e., participants' demographic data, current professional roles, study's objective, informed consent, and general opinion). The general interview questions focused more on the activities, performance, resources, and barriers to NGO participation in moving toward UHC. It was validated by three NGO representatives and two experts for face and content validation and piloted under intended interview conditions to confirm the validity and practice for the interviewer. The data on these pilot interviews were also used in the study analysis. An independent interviewer conducted all interviews. The research team consisted of four academic researchers, three male researchers and one female researcher. All four research team members have prior experience in qualitative research. All participants provided written informed consent before participating in interviews. With participants' consent, all conducted interviews were audio-recorded, each lasting on average 38 min. A total of 33 interviews were conducted with the research participants.

### Document review

Document reviews were used as Supplementary Data. To conduct document reviews, relevant documents were collected from universities of medical sciences, MoHME, and local websites, such as registered documents in the “database for SAMANs.” Data on NGOs' activities, their population coverage, provided services and the costs from NGOs' financial records, strategic and operational plans, minutes of meeting between NGOs and authorities of vice-chancellors of social affairs, and other related documents were collected.

### Data analysis

All interviews were transcribed verbatim. Analyses were performed simultaneously with transcription. If there were any inconsistencies or ambiguities, these were resolved by calling back interviewees for clarifications. To ensure the data were robust, we used strategies of member checking (in three stages of data collection, at the end of each interview, and after data analyses) and auditing (after extracting initial codes and development of initial themes). The interviews and policy documents were analyzed by the content analysis method. MAXQDA 12 (VERBI Software 2015, MAXQDA 12, computer program, VERBI Software, Berlin) was used for data analyses. Data coding process was done in three steps: generating and finalizing the initial codes, grouping codes denoting the same concepts into subcategories, and classifying subcategories indicating similar subjects into the main categories. Two authors read all documents and transcripts of the interviews to extract issues, themes, and sub-themes. During the data analysis phase, the two researchers had regular meetings to discuss any disagreements to reach a consensus openly. When consensus was not reached, the other two researchers resolved any disputes.

## Results

Based on the thematic analyses of interviews and document reviews, nine main themes and one hundred and five sub-themes were identified. Each theme was categorized based on three factors: NGO-, society-, and government-related factors (Table 2). The strategies and challenges of each theme, if any, were stated in its sub-themes.

**Table 2:** Themes and sub-themes.

**Main themes**	**NGOs related factors**	**Society related factors**	**Government-related factors**
Services coverage	Coverage of treatment services. Coverage of prevention services. Coverage of rehabilitation services.	Involving the society in improving health.?	Reinforce and institutionalize intersectoral cooperation. Training and empowering NGOs and strengthening their skills.
Population coverage	Coverage of specific patients. Coverage of infectious patients. Coverage of vulnerable groups.	Collaborate with community members to identify people eligible to receive services from NGOs. Introducing areas with low levels of health facilities by community members to NGOs.	Collaborate with government and government agencies to identify people eligible to receive services from NGOs.
Financial coverage	Coverage of livelihood expenses. Economic assistance. Reducing medical costs.	Taxes paid by the people. Donate people to NGOs.	Government subsidies to NGOs for pharmaceutical services. Financial support for health NGOs.
Quality of services	Providing health facilities. Providing health equipment. Increasing public satisfaction. Increasing the health workforce. Improving the quality of health services for the community.	Participate in the promotion of equitable health. Exchange of information between health care organizations and the public. Increase people's responsibility and improve their efficiency in the field of health. Strengthen people's participation in improving the quality of services.	Social capital development Encourage and prepare NGOs.
Participating in policymaking	Having interactive communication and regular meetings between NGOs. Attendance and presentation of the p~rogram by NGOs in policymaking meetings. A commitment of members to NGO activities. Being strategic of NGOs activities in UHC. Presenting their plans and capabilities to policymakers and the government. Being the voice of people.	Acceptance of NGOs by society. Trust in NGOs by the community Interaction and cooperation of the community with NGOs.	Acceptance of NGOs. Trust in NGOs. Creating communication channels formal with NGOs Inviting NGOs to UHC-related meetings. Defining the role of NGOs in the goals of UHC. Transparency of UHC-related programs, rules and policies. Organizing the status of donors. Creating a positive and strong belief in the government about the role of NGOs in UHC. Creating transparency in the rules related to health NGOs.
Participate in the implementation of policies and programs	Participation in government projects. Advising on UHC projects. Provide competent human resources to the government in advancing the goals of UHC. Giving practical motivation to the human resources of NGOs. Avoid doing parallel work to UHC. Having sufficient cooperation between NGOs and donors. Creating specific goals and policies among the NGOs to move toward UHC. Resource mobilization.	Supportive of NGOs. Joining community members as volunteers to NGOs. Establishing communication with NGOs. Participation of community members with NGOs.	Following up the problems of NGOs in the field of UHC. Provide sufficient time to express the challenges of NGOs active in UHC. Giving practical incentives to NGOs to advance the goals of UHC. Introducing areas that need more participation. Outsourcing activities to NGOs. Giving responsibility to activists active in the field of UHC.
Culture building and support	Creating a sense of participation in people. Supporting your target community. Creating a culture of evaluation and public opinion in relation to programs and activities related to UHC. Seeking support to change specific laws and policies in favor of UHC.	Encouraging benevolent culture by the people. Recognizing NGOs as a bridge. between society and the government. Creating a culture of cooperation of community members with NGOs in order to educate, empower and involve the community. Increasing public awareness of the benefits of participating in NGOs.	Promoting a culture of benevolence among members of the community. Creating a sense of participation in people. The relationship between NGOs and the government. Perform symbolic gestures.
Monitoring and evaluation	Litigation in judicial and quasi-judicial authorities to assert the health rights of the target community. Report deficiencies related to UHC programs. Spontaneous monitoring of programs and activities related to UHC. Allocating the supervisory force in NGOs. Monitoring the cost of activities related to UHC.	Spontaneous monitoring of people on NGOs. Reporting possible violations of NGOs to the relevant devices. Asking people for transparency in financial and non-financial assistance to their target community.	Creating legal cases for the duties, rights, supervision and violations of UHC. Creating an evaluation model of active NGOs in UHC. Systematic monitoring of active NGOs in the field of UHC. Creating and defining a monitoring system. Participating in government projects as an observer or consultant.
financing NGOs	Managing financial resources. Generating new financial resources for UHC. Covering the population who do not have basic health insurance. Managing financial protection and providing service to those with life-threatening and incurable diseases. Creating a comprehensive and strategic plan in financing. Create a specific program in financial assistance to the target community. Creating transparency in financial aid and donors. Proper financial management. Encourage members to donate voluntarily.	Financial assistance to NGOs from the community. Encourage community members to donate voluntarily.	Provide Sustainable financial resources for NGOs. Allocation of special taxes. Allocating targeted financial resources to NGOs.

Based on the review of documents and the interviews, NGOs in Iran primarily had activities in ten health-related areas, including healthcare with five sub-sectors (public health development, environmental health, medicine, patient treatment and relief, and health infrastructure). The activity license of NGOs at national and provincial levels is issued through the Ministry of Interior, the Governor and Governorates and State Welfare Organization of Iran, the police force of the Islamic Republic of Iran, and the Ministry of Sports and Youth. Hence, NGOs can be differentiated based on their characteristics, that is, non-governmental, non-profit, non-political, and operating at national, regional, provincial, or international levels. In some Iranian national upstream documents, the importance of NGOs has been mentioned. NGOs working in the public health had six general programs, including training and empowerment of NGOs with the help of university or faculty of medical sciences through holding educational workshops, university and faculty of medical sciences' support of NGOs in line with the general health goals announced by the Minister of Health and Medical Education (MoHME), monitoring and evaluation of NGOs with the help of university and faculty of medical sciences, expanding the activities of national NGOs to the provinces and creating NGOs in provinces, and building networks in the provincial or national level in subjects such as AIDS or addiction.

### NGOs' performance and UHC

There were conflicting views on the performance of NGOs in achieving the goals of moving toward UHC. NGO staff believed that their performance positively affected public health and health outcomes. Some participants mentioned that evaluation of the performance of NGOs and its effect on the health system is not possible. Lack of definition of NGOs' role and expectations of NGOs in line with UHC programs and the lack of an effective evaluation program of the activities of these organizations in achieving the goals of UHC were among the challenges mentioned by participants.

“*When we can discuss the role of NGOs in universal health coverage and say it is good or bad, first of all, we should have a tool that would allow evaluating how they work and secondly what do we expect from NGOs in public health coverage.” [A NGOs researcher]*

However, the establishment of the National Health Assembly with the slogan “All for health, health for all” in line with the Health Transformation Plan in Iran is a turning point in the participation of NGOs in UHC-related policies.

### Service coverage

One of the factors influencing NGOs' participation in achieving UHC was service coverage. NGOs provided different services in three levels in this theme—prevention, treatment, and rehabilitation. Both NGO staff and experts agreed that the most NGO activity was in service coverage.

“*In general, NGOs work more in the field of service coverage than other dimensions because it is more tangible and has better facilities in this area*.” *[A manager at NGO]*

NGO activities in the field of prevention services were related to education (such as obesity, overweight, high fat, high blood sugar, and high blood pressure and its effect on cardiovascular diseases, and healthy life) and awareness (adhering to a healthy diet, not smoking, and inactivity), empowerment, screening for communicable/non-communicable diseases, disseminating health-oriented information, and promoting community health. Also, other activities by NGOs were to cover services: the provision of curative services, which includes periodic visits by a general practitioner, the provision of dental services, and specialized services to patients according to their illness. Some NGOs performed activities in the coverage of rehabilitation services, including therapeutic counseling, occupational therapy, and speech therapy. The activities of NGOs in the field of treatment were mostly focused on providing medical services freely or with low cost to patients with special diseases such as diabetes, cancers, and blood diseases.

“*NGOs have a variety of activities including education, community empowerment, dental services, chemotherapy services, counselling services...” [A head of Licensing Unit to NGOs]*

On the contrary, one of the strategies that NGOs should consider and in which the community is involved is to involve the general public in improving health issues. Also, one of the challenges of the government in covering services is the issue of inter-sectoral cooperation, especially cooperation with NGOs, which the government should strengthen this area and adopt an appropriate strategy for it.

“*NGOs can also count on people's capacity and ability in this field” [A manager at NGO]*. Also, another participant stated, “*In my opinion, the government is not fully utilising the capacity of NGOs and should increase its cooperation.” [An NGO staff]*

### Population coverage

One of the main foci of work NGOs was increasing population access to health services, particularly for vulnerable, poor, or people with a specific disease, using strategies and interventions such as community participation. Coverage of patients with particular conditions, infectious diseases, and coverage of vulnerable groups were among the main population covered by the NGOs. A review of documents showed that most of the NGOs in the public health field covered people with specific diseases such as kidney, cancer, hemophilia, thalassemia, and phenylketonuria. Another measure taken by NGOs to increase population coverage was to cover people with infectious diseases, such as leprosy, tuberculosis, and AIDS. Also, NGOs had a significant role in identifying vulnerable groups, such as marginalized, displaced, and informal populations, and providing health services to these populations.

“*The role that NGOs can play in population coverage is to cover informal and marginalised populations such as immigrants, sex workers, etc. This population is not formally covered by insurance. We should consider that identifying this population is difficult. The role of NGOs is very prominent in identifying and introducing this population because they are located in the most peripheral part of the county.” [A senior health official]*

The role of society is also significant, and since NGOs alone are not able to identify people and areas with poor health, people should aid NGOs and work with them. The government should also ask government agencies operating in health to cooperate with NGOs to identify people who are not in good health status.

“*The people and the government can identify areas where NGOs need to cooperate so that they can go there and volunteer.” [A manager at NGO]*

### Financial coverage

According to the participants and document review, NGOs used various strategies to provide financial coverage to their populations. These strategies included coverage of livelihood expenses, economic assistance, and reducing medical costs. According to NGO mission, some tried to cover the living expenses of the people under their coverage by providing marriage, education, livelihood assistance, and travel allowances. The most critical action that NGOs took in UHC was reducing expenses by covering medical and pharmaceutical expenses in whole or in part. Also, another activity of NGOs in Iran was related to cooperation with local institutions that provide loans to people in the community to cover part of their health expenses.

“*We provide aid in the form of a package or financial aid to people that might need any care or are in poverty or at risk of catastrophic health expenditure situation*.” *[A manager at NGO]*

None of the NGOs had a strategy in line with the insurance coverage plans. They reduce the health costs of the covered people through direct payment of medical and pharmaceutical costs and also by making contracts with clinics and hospitals to provide services with low cost.

### Quality of services

NGOs also worked toward improving the quality of services. In this regard, provision of health facilities, establishing medical centers or hospitals with donors' help, and equipping medical centers were among other activities mentioned by participants.

“*Our NGO bought a new device for the hospital with the help of donors…. providing equipment such as condoms, wheelchairs and other medical needs of the population are some of the other activities that we do in our NGO.” [A manager at NGO]*

Participant also stated that by recruiting volunteers and health workers, who are the official forces of the Ministry of Health, NGOs increased the number of services provided during non-operating hours in clinics and hospitals. By recruiting staff volunteers, they also increased the active workforce and improved the quality of services offered in health, which led to an increase in public satisfaction.

“*NGOs can, with the help of specialist physicians and nurses, ask them to volunteer at NGO centres during the week.” [A NGOs researcher]*

Factors affecting the participation of NGOs in the community in providing quality of services included involvement in the promotion of equitable health, exchange of information between healthcare organizations and the public people, increase people's responsibility, and improving their efficiency in the field of health and strengthening people's participation in improving the quality of services. On the contrary, the government promoted social capital and ultimately increased the quality of services by encouraging and preparing NGOs.

“*I think people have a vital role to play in the quality of services provided by NGOs, and they should be involved in public health.” [A head of Licensing Unit to NGOs]*

### Participating in policymaking

According to NGO-related factors, having interactive communication and regular meetings between NGOs, attendance, and presentation of a program by NGOs in policymaking meetings, the commitment of members to NGO activities, being strategic of NGOs activities in UHC, presenting their plans and capabilities to policymakers and the government, and being the voice of people would lead to increased engagement of NGOs in the field of UHC.

“*We must be active in policy-making meetings and be able to introduce ourselves properly.” [A manager at NGO]*

In addition, acceptance of NGOs by society, trust in NGOs by the community, and interaction and cooperation of the community with NGOs were identified as crucial factors that can lead to active participation from NGOs in UHC. Finally, acceptance of NGOs, trust in NGOs, creating communication channels formal with NGOs, inviting NGOs to UHC-related meetings, defining the role of NGOs in the goals of UHC, transparency of UHC-related programs, rules, and policies, organizing the status of donors, creating a positive and strong belief in the government about the role of NGOs in UHC, and creating transparency in the rules related to health NGOs by the government led to increased engagement of NGOs.

“*There must be a strong will in the government to involve NGOs.” [A NGOs researcher]* Also, another participant stated, “*People need to trust NGOs and work together.” [A NGOs' staff]*

### Participate in the implementation of policies and programs

Participants mentioned that NGOs could assist the government in implementing programs and procedures by focusing and participating in government projects in UHC, providing qualified human resources and motivating volunteer forces, and mobilizing resources.

“*We must have a competent force to carry out so that the government can trust us and give us some of its activities.” [A board of directors of NGO]*

The community could implement UHC-related programs and policies by connecting and partnering with NGOs. In addition, the government could encourage NGOs to participate and address their problems by outsourcing some activities and creating more participation and enough time to implement programs and motivate NGOs.

“*Our society has a lot of educated people, and when they see that they can help, they should come to work and help NGOs*.” *[A manager at NGO]*

The participants stated that the country's health management system should give priority to delegation of authority and responsibility to the actors in the field of health, and in this regard, the National Health Assembly can be an effective consultative organization.

“*… The formation of this assembly is emphasized as an effective advisory arm for the Supreme Council of Health and Food Safety.” [A manager at NGO]*

### Culture building and support

The study participants expected that NGOs would promote a culture of participation among the people, the government, and organizations to support their target community and support UHC-related laws and policies or seek to change them.

“*The media can teach a culture of benevolence in the country and introduce NGOs to the people.” [A NGOs researcher]*

In government and community-related factors, participants provided information about encouraging the community to help and understanding that NGOs are a bridge between community and government.

“*Unfortunately, in the last 10, 15 years, they gave false information about NGOs to the people, but during these years, NGOs tried to show their true face to the people and the government, and we see that trust in them has increased.” [A manager at NGO]*

### Monitoring and evaluation

In this regard, participants stated that NGOs should cooperate with the relevant judiciary and executive bodies in UHC and report any violations in the case.

“*This year, the judiciary has designed a platform on which NGOs can voluntarily register, which I think is a good activity.” [A board of directors of NGO]*

In addition, society can help monitor and evaluate NGOs in UHC-related programs through voluntary monitoring, reporting of potential violations by NGOs, and transparency in funding. The government should strengthen the monitoring and evaluation system by establishing and defining a monitoring system, reviewing the principles of NGOs, and using NGOs as supervisors or consulting in UHC-related projects.

“*One of the capabilities that I think has been overlooked by the government is that NGOs can supervisors' projects.” [A senior health official]*

### Financing NGOs

Participants stated that financing is one of the critical issues regarding the participation of NGOs in UHC programs and stressed that managing financial resources, generating new financial resources for UHC, covering the population who do not have basic health insurance, managing financial protection and providing service to those with life-threatening and incurable diseases, creating a comprehensive and strategic plan in the financing, creating a specific program in financial assistance to the target community, creating transparency in financial aid and donors, proper financial management, and encouraging members to donate voluntarily are essential. In addition, they acknowledged that the government could assist them by providing sustainable financial resources for NGOs, allocating special taxes, and providing targeted financial resources to NGOs.

“*I have seldom noticed that NGOs or the government have a comprehensive funding program organisation, which I think can follow the example of successful countries like Italy, which levies part of the tax and allocates it to NGOs.” [An NGO member]*

At the same time, due to Iran's economic conditions and external sanctions during the years, most of the funding sources of NGOs through foreign donors and international organizations were reduced.

“*Due to the economic war with the world and sanctions, it is impossible to use the capacity of foreign aid, and unfortunately during last year's NGOs suffered a lot from this issue, and it affected their revenues and costs, and now NGOs are more focused on internal resources.” [A manager at NGO]*

## Discussion

This study examined the viewpoints of scientific experts and stakeholders on NGO UHC-related activities in Iran. Our results showed that NGOs work in all UHC dimensions in Iran, including quality service coverage, population coverage, and financial coverage. One of the significant issues raised was that the government has not appropriately used its full potential. Our findings also imply that lack of programs in this regard, lack of tools (including appropriate criteria for evaluating actions and activities of the NGOs), and ambiguity in the role and position of NGOs in the health system structure are among the main challenges of using NGOs potential in moving toward UHC in Iran to the fullest. For years, NGOs have tried to fill the gaps in health services provision by MoHME. They are increasingly stepping up along with healthcare providers and pursuing similar goals. This is confirmed by previous studies showing that the government's lack of a defined plan and resource constraints limits their participation in health system goals. Evidence shows that achieving social health is not possible without the participation of NGOs, especially in low-income countries (24). Nonetheless, NGO participation in healthcare is recognized as an effective strategy for promoting health, social development, and access to UHC.

NGOs can provide services directly through screening for communicable or non-communicable diseases, disseminating health information, equipping and developing infrastructure of healthcare centers, or indirectly through counseling and supporting services in prevention (24). They can also provide follow-ups and a wide range of pharmaceutical, outpatient and inpatient, and diagnostic services directly by covering drug and medical expenses or indirectly providing in-home support services (25–27). However, for better NGOs contribution and improving their motivation to participate in health system activities, the costs of NGOs need to be covered by supranational organizations such as the State Welfare Organization of Iran and the Relief Committee, or indirectly through contracts with medical centers. Studies conducted in other developing countries showed that NGOs could provide structural services such as building hospitals and providing medical equipment (15, 28–30). Other evidence showed that NGOs could improve and promote the health of their communities through the establishment of primary healthcare centers, providing laboratory services, training community health workers to screen for and manage chronic hypertension, providing maternal and new-born health services, providing medical services for children with cancers, providing mental health services through community-based rehabilitation, prevention and treatment groups received growth monitoring, referrals to public health facilities, home-based counseling, and providing mid-day meals for primary school students and adolescents (31–40).

In many countries, the poor still have limited access to primary healthcare, and NGOs can increase access to health services because of their ability to design population-based projects. NGOs are also able to implement prevention programs to reach vulnerable social populations. Innovative approaches such as the caregiver approach can be a promising alternative to existing strategies to provide critical healthcare to disadvantaged communities (41–44). UHC is a key priority set out by the WHO and the United Nations General Assembly (45, 46). Social health insurance schemes, one mechanism to achieve UHC, have become increasingly crucial in low- and middle-income countries as they work to achieve this goal. To ensure comprehensive health insurance coverage for a broad population at a reasonable cost, social health insurance schemes could be designed by NGOs so that individuals receive a set package of subsidized health services through accredited providers (45, 47, 48). NGOs can rightly play a vital role in moving toward UHC when their role is defined in UHC programs by the government and the Ministry of Health or other relevant entities. Therefore, health policymakers and planners should facilitate adequate and targeted services by NGOs by prioritizing important components of UHC and the goals required for medical and educational interventions (24).

In line with our findings, a recent study conducted in Iran showed that legal and managerial barriers are among challenges for NGOs to work with the government to move toward UHC (16). NGOs need to use appropriate strategies to achieve UHC goals. As noted in our study, building sustainable funding and developing strategies for the participation in legislative, planning, and decision-making levels are critical. For example, designing and implementing programs and policies related to health system goals and NGOs should be conducted with the support of the community and people who specialize in this field (9). The government should also create the right opportunities for NGOs to participate in public health and UHC activities, use them as advisors, and delegate responsibilities because these organizations have a very close relationship with the community. They are known as the voice of the people. In terms of the partnership of these organizations with the government and the capacity on both sides (both the government and the NGOs), the community and their participation are very effective. Previous studies in this area were also consistent with the findings of our study (49–55).

Collaboration with government and other government agencies is a key strategy for NGOs moving toward UHC, and this creates synergies and uses their capabilities. In other words, as it was shown in our study, NGOs need to formally communicate with all institutions involved in UHC to provide quality services to people in the community, especially vulnerable and marginalized groups (56, 57). At the same time, creating a human culture encouraging community members to help NGOs and encouraging a sense of participation in the community causes significant cooperation between the government, government agencies, and the community with NGOs (15).

## Conclusion

NGOs could have a significant role in providing each UHC dimension and could have great potential in moving countries toward UHC. However, to use the NGOs potential, the government and MoHME must define the scope of NGOs activities and develop a comprehensive plan for engaging NGOs in UHC. Given the creation and expansion of health services and global attention to UHC, NGOs' participation can improve services provided to the populations, particularly the poor and marginalized areas. Despite the vital role of NGOs in health services delivery, relatively little is still known about how NGOs could be engaged to achieve UHC goals. NGOs' goals and expectations of public health coverage programs in Iran are not clear. Understanding NGOs' role and contributing to attaining UHC is critical, especially in the local context. Governments need to consider developing systematic and fundamental strategies for NGOs engagement in public health goals to move toward UHC.

## Data availability statement

The raw data supporting the conclusions of this article will be made available by the authors, without undue reservation.

## Ethics statement

This study was approved by the Ethics Committee of Tabriz University of Medical Sciences (Approval No: IR.TBZMED.REC.1399.370). The patients/participants provided their written informed consent to participate in this study.

## Author contributions

LD conceived the study. AS and LD contributed to designing, collecting, analyzing, drafting, and finalizing the paper. RK-Z and VS contributed to analyzing data. All authors read and approved the final version of the paper. All authors contributed to the study conception and design.

## Funding

The study was funded by Tabriz University of Medical Sciences, Tabriz, Iran (Grant No: 64241).

## Conflict of interest

The authors declare that the research was conducted in the absence of any commercial or financial relationships that could be construed as a potential conflict of interest.

## Publisher's note

All claims expressed in this article are solely those of the authors and do not necessarily represent those of their affiliated organizations, or those of the publisher, the editors and the reviewers. Any product that may be evaluated in this article, or claim that may be made by its manufacturer, is not guaranteed or endorsed by the publisher.

## Abbreviations

NGOs: non-governmental organizations
UHC: universal health coverage
MS: multiple sclerosis
PKU: phenylketonuria.
